# Current Strategies for Selecting Recombinant Orthopoxvirus Proteins for Immunobiological and Diagnostic Applications

**DOI:** 10.3390/v18080863

**Published:** 2026-08-07

**Authors:** Aigerim Zhakypbek, Lespek Kutumbetov, Gulnur Kuzembekova, Kamshat Shorayeva, Nurlan Kozhabergenov, Bekbolat Usserbayev, Gulnur Nakhanova, Kuanysh Jekebekov, Gaukhar Shynybekova, Akbope Abdykalyk, Aknur Ulankyzy, Temirlan Baiseit, Balzhan Myrzakhmetova, Olga Chervyakova, Kuandyk Zhugunissov

**Affiliations:** 1 Research Institute for Biological Safety Problems, National Holding QazBioPharm, Gvardeiskiy 080409, Kazakhstan; a.zhakypbek@biosafety.kz (A.Z.);; 2 Department of Microbiology, Virology and Immunology, Faculty of Veterinary Science and Zooengineering, Kazakh National Agrarian Research University, Almaty 050010, Kazakhstan

**Keywords:** orthopoxvirus, monkeypox virus, recombinant antigens, serological diagnostics, subunit vaccines, IMV and EEV antigens, phylogenetic analysis, immunogenicity

## Abstract

Orthopoxviruses are zoonotic DNA viruses of continuing public health importance, as highlighted by recent global mpox outbreaks. The high antigenic similarity among orthopoxviruses enables cross-reactive immune responses but complicates the development of serological diagnostic systems for serosurveillance, assessment of previous exposure, evaluation of vaccine-induced immunity, and species differentiation. This review summarizes current knowledge regarding the structure, biological functions, and diagnostic and vaccine potential of major orthopoxvirus recombinant antigens. Particular attention is given to the genomic organization of *Orthopoxvirus*, differences between intracellular mature virion (IMV) and extracellular enveloped virion (EV) forms, and the characteristics of key antigens, including A29, M1, H3, A35, and B6. Comparative phylogenetic analyses demonstrated a high degree of conservation among structurally important orthopoxvirus proteins while also identifying variable regions potentially relevant for differential diagnostics. Available evidence indicates that IMV-associated proteins are primarily involved in virus neutralization, whereas EV-associated antigens contribute mainly to limiting viral dissemination. Consequently, multicomponent antigen strategies combining IMV- and EV-associated proteins appear to provide the greatest potential for the development of effective serological assays and next-generation subunit vaccines. Overall, rational antigen selection should be based on integrated evaluation of structural, functional, and immunological properties to optimize orthopoxvirus diagnostic and vaccine platforms.

## 1. Introduction

The genus *Orthopoxvirus* belongs to the subfamily *Chordopoxvirinae* within the family *Poxviridae* and comprises large double-stranded DNA viruses characterized by a highly conserved genomic architecture [1,2]. Among members of the *Poxviridae* family, orthopoxviruses are the most extensively studied because of their considerable medical importance. According to the latest taxonomy release of the International Committee on Taxonomy of Viruses (ICTV, Virus Taxonomy Release 2023), the genus currently includes 12 officially recognized species. Of these, variola virus, monkeypox virus, cowpox virus, and vaccinia virus are considered the most clinically significant due to their ability to infect humans and cause clinically manifested disease [3].

Long before the emergence of the global mpox outbreak in 2022, orthopoxviruses had been extensively studied as important human and veterinary pathogens. Research on variola virus, vaccinia virus, and cowpox virus provided fundamental insights into orthopoxvirus biology, immune responses, mechanisms of protection, and antigenic targets. Many of the proteins currently investigated for mpox vaccines and serological diagnostics were initially identified and characterized in studies using vaccinia virus and other orthopoxvirus species, forming the basis for contemporary mpox research [4,5,6].

Between 2022 and 2025, more than 140,000 confirmed mpox cases were reported worldwide. The global mpox epidemic that began in 2022 was predominantly associated with MPXV clade IIb, particularly lineage B.1, whereas recent transmission events in endemic African regions have involved clade I viruses, including clades Ia and Ib [7]. During 2024–2025, sustained transmission was observed predominantly in African countries, reflecting the continued epidemiological activity of the virus in endemic regions [7]. At the same time, cases continued to emerge outside endemic areas, including in previously non-endemic regions across multiple continents. In 2025, locally acquired infections caused by MPXV clade I were reported in several EU/EEA countries, including Spain, Italy, the Netherlands, and Portugal, while ongoing regional transmission was also documented in parts of the United States [8]. These outbreaks beyond endemic settings further emphasize the persistent public health relevance of orthopoxviruses and highlight the need for improved preventive and diagnostic strategies, including the development of effective vaccines and highly specific serological assays.

Despite their historically established names, which often reflect presumed animal hosts, most members of the genus *Orthopoxvirus* possess a substantially broader host range, primarily involving small mammals. This broad host tropism underlies their pronounced zoonotic potential and their considerable importance in veterinary virology [9]. For example, despite its designation, cowpox virus is endemic mainly among wild rodent populations in Europe, particularly voles and woodland mice, whereas cattle and humans represent incidental hosts, illustrating both the zoonotic nature of the infection and the conditional nature of orthopoxvirus nomenclature [10]. Field studies conducted in endemic regions of West Africa have identified molecular and serological evidence of orthopoxvirus circulation in multiple small rodent species, including both sylvatic and peridomestic animals, supporting the existence of a multi-host reservoir system and several potential routes of zoonotic transmission [11]. Collectively, current evidence on the broad host spectrum and geographic distribution of orthopoxviruses suggests that the diversity of *Orthopoxvirus* species and their natural reservoirs contributes to the formation of a heterogeneous antigenic landscape. As summarized in Table 1, members of the genus are predominantly associated with rodent hosts, which largely determines the long-term ecological maintenance and epidemiological persistence of these viruses [12].

Collectively, available evidence on the diversity of natural reservoirs and host species indicates that most orthopoxviruses are maintained through long-term ecological circulation within natural ecosystems, primarily among small mammals, without establishing sustained chains of human-to-human transmission. Under these conditions, infections in domestic animals and humans occur sporadically as zoonotic spillover events and are generally associated with direct contact, accidental exposure outside stable transmission cycles, and various anthropogenic factors [40].

**Figure 1:**
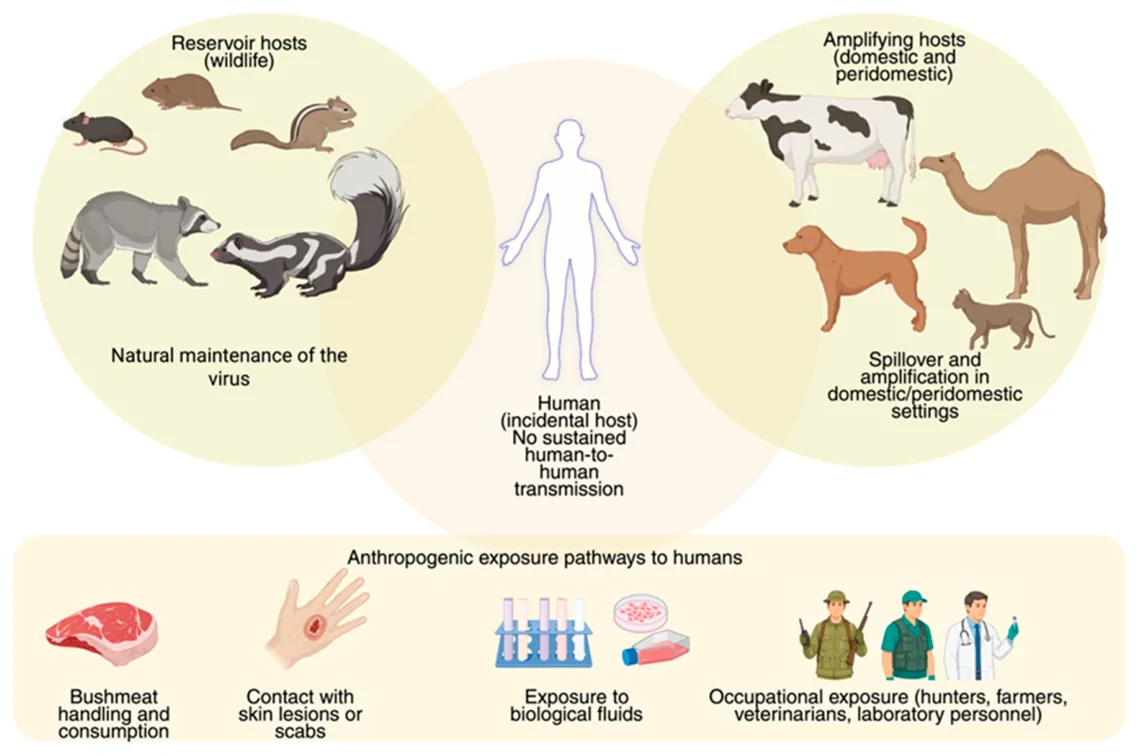
*Orthopoxvirus* transmission model. The diagram illustrates the ecological transmission model of orthopoxviruses, highlighting confirmed and suspected wildlife reservoirs, viral transmission to domestic and peridomestic animals, and humans as incidental hosts. Anthropogenic routes of infection reflect behavioral and occupational risk factors rather than sustained transmission cycles and emphasize the ecological context underlying antigen conservation and cross-reactive immune responses.

This ecological pattern of circulation (Figure 1) is associated with a relatively stable immunological landscape, which is consistent with the conserved antigenic structure of major orthopoxvirus proteins and the pronounced cross-serological reactivity observed within the genus *Orthopoxvirus*, as demonstrated by both serological and molecular studies [41,42]. A representative example of such spillover transmission was reported in France, where cowpox virus infection was transmitted from pet rats to humans through direct contact without subsequent human-to-human spread, illustrating the opportunistic nature of orthopoxvirus zoonotic transmission [43].

In this context, the selection of antigens for serological diagnostics requires a balanced approach that considers both the extensive cross-reactive immune responses responsible for the high sensitivity of orthopoxvirus test systems and the presence of variable epitopes that may enhance diagnostic specificity.

A characteristic feature of the genus *Orthopoxvirus* is the high degree of antigenic conservation, which underlies pronounced immunological cross-reactivity and the development of cross-protective immune responses against infections caused by different orthopoxvirus species [44,45]. Consequently, detailed characterization of individual orthopoxvirus proteins is of critical importance for the rational selection of recombinant antigens used in serological diagnostic platforms.

Throughout this review, MPXV protein nomenclature is used for orthologous orthopoxvirus proteins. Where appropriate, the corresponding VACV orthologs are indicated, as many experimental studies have been conducted using VACV orthologs.

## 2. Genomic Organization of Orthopoxviruses and the Antigenic Landscape

*Orthopoxvirus* genomes consist of linear double-stranded DNA molecules approximately 200 kb in length. Their termini are covalently closed, forming a continuous polynucleotide structure, a characteristic feature of members of the family *Poxviridae* that distinguishes them from most other DNA viruses [46,47]. The viral genome encodes approximately 200 proteins responsible for the structural and functional organization of the virion.

Several major groups of proteins can be distinguished within the orthopoxvirus virion according to their localization and biological functions. These include proteins associated with the intracellular mature virion (IMV), proteins specific to the extracellular enveloped virion (EV), as well as proteins involved in viral entry and virion egress from infected cells [47].

Comparative genomic analyses of orthopoxviruses demonstrate a pronounced functional stratification of the viral genome. Studies of the attenuated Modified vaccinia Ankara (MVA) strain have shown that the major genomic alterations are concentrated predominantly within terminal regions encoding host interaction factors, whereas the central genomic region remains relatively conserved [48,49]. Structurally, orthopoxvirus genomes are organized into a conserved central region and variable terminal domains. The central genomic region contains a highly conserved set of orthologous genes shared among most members of the genus *Orthopoxvirus* and encodes proteins essential for viral replication, virion morphogenesis, and the formation of core antigenic structures [50,51].

In contrast, the terminal regions of the orthopoxvirus genome are highly variable and encode genes involved in host range determination and immune modulation. Approximately 105 accessory genes have been described within the genus *Orthopoxvirus*, although their composition differs substantially among species. The most extensive repertoire of accessory genes has been identified in certain cowpox virus strains, whereas variola virus and monkeypox virus genomes contain considerably reduced sets of these genes, reflecting distinct evolutionary strategies of host adaptation [52,53].

The variability of the accessory genomic repertoire is driven mainly by gene loss and duplication events during orthopoxvirus evolution and is concentrated within terminal genomic regions, including the inverted terminal repeats (ITRs), while the central genomic region remains highly conserved. Comparative pangenomic analyses indicate that accessory genes may account for up to 40–50% of the orthopoxvirus genome, whereas the high frequency of deletions, duplications, and gene loss events in terminal regions contributes substantially to interspecies and interstrain differences in immunomodulatory protein repertoires [50,51].

This genomic organization contributes to the biological diversity and host adaptation observed among orthopoxvirus species [53,54]. Although orthopoxviruses share a highly conserved genomic architecture and substantial antigenic similarity, the degree of genetic diversity differs considerably among species. Variola virus and monkeypox virus are relatively homogeneous lineages, whereas cowpox virus exhibits substantially greater genetic diversity and represents one of the most heterogeneous members of the genus. Consequently, conclusions regarding antigen conservation and serological cross-reactivity should be interpreted with consideration of species-specific evolutionary diversity. While this review considers the *Orthopoxvirus* genus as a whole, the majority of studies discussed in this review focus on variola virus, vaccinia virus, monkeypox virus, and cowpox virus, as these species remain the most extensively investigated members of the genus [50,51,52]. Alterations within terminal genomic regions are frequently associated with differences in pathogenicity, host range, and immunogenicity between naturally circulating and attenuated orthopoxviruses [50,51,52,55].

Orthopoxviruses exist as two antigenically distinct virion forms: the mature virion (MV) and the extracellular enveloped virion (EV). Cross-protective immunity among orthopoxvirus species is largely associated with the high conservation of major surface proteins and with the fact that effective antibody responses are directed against multiple antigens derived from both virion forms [56].

Despite the high amino acid conservation of key orthopoxvirus antigens, cross-serological reactivity does not always correlate with efficient viral neutralization. This discrepancy is associated with antigenic distances between viruses, as well as differences in the composition, accessibility, and immunodominance of neutralizing epitopes [57]. In contrast to vaccinia virus, variola virus strains, including Alastrim (variola minor), exhibit high levels of genetic and phenotypic stability and retain their biological properties during serial passage in different host systems, indicating substantial differences in genomic plasticity among orthopoxviruses [58].

Comparative genomic analyses of Old World orthopoxviruses have demonstrated that, in addition to E3 and K3, these viruses encode several other conserved proteins involved in suppression of innate immune responses, suggesting functional redundancy among viral immune evasion mechanisms [44]. The evolution of variola virus was accompanied by extensive gene loss, comparable to the genomic reduction observed in monkeypox virus [57].

Recent in silico studies have demonstrated that several proteins involved in viral entry and virion egress retain a high degree of amino acid conservation, including in recently emerged monkeypox virus lineages, highlighting their potential as targets for the induction of cross-protective immune responses [59]. One example of such conserved antigens is the MPXV A28 (VACV A26; OPG153) protein, which has been shown to induce broadly neutralizing antibodies displaying cross-reactivity against multiple MPXV clades, vaccinia virus, and orthologous proteins of other orthopoxviruses [60].

To illustrate the phylogenetic relationships within the genus *Orthopoxvirus*, complete genome sequences of representative viruses were retrieved from the GenBank database. A total of 12 genome sequences representing nine *Orthopoxvirus* species were selected (Table 2). Phylogenetic analyses were subsequently performed using orthologous sequences of the selected antigen genes.

Amino acid sequence alignment and phylogenetic tree reconstruction were performed using MEGA11 software. Phylogenetic trees were constructed using the Neighbor-Joining method with Poisson-corrected amino acid distances. The robustness of the tree topology was assessed by bootstrap analysis with 1000 replicates. Evolutionary distances were expressed as the number of amino acid substitutions per site [61].

## 3. Recombinant Proteins as Tools for the Development of Modern Diagnostic Platforms and Vaccines

In the current literature, protein subunit vaccines against orthopoxviruses are regarded as a safer alternative to live vaccines. However, despite their favorable safety profile, they generally exhibit lower intrinsic immunogenicity and therefore often require adjuvants, booster immunizations, and carefully selected antigen combinations to achieve robust protective immunity [4,5,6,59,62]. Several vaccine platforms have been investigated for the prevention of mpox and other orthopoxvirus infections, including live attenuated, DNA, mRNA, and recombinant protein-based vaccines. Live attenuated vaccines provide broad humoral and cellular immune responses and currently represent the only licensed approach for mpox prevention. However, the safety profiles of currently available live vaccinia-based vaccines differ substantially. Replication-competent vaccinia vaccines, such as ACAM2000, have been associated with serious adverse events, including myopericarditis [63]. In contrast, Modified vaccinia Ankara (MVA) is a replication-deficient vaccine that does not produce infectious virus in human cells, although viral gene expression occurs following infection [64]. DNA vaccines have demonstrated protective efficacy against lethal monkeypox challenge in non-human primates, while more recent mRNA vaccine candidates expressing multiple MPXV antigens have elicited robust humoral and cellular immune responses in animal models [65,66,67]. According to data from U.S. regulatory agencies, two live vaccines are currently approved for the prevention of smallpox and mpox, whereas no licensed protein-based vaccines are presently available, and most candidate formulations remain at the preclinical stage of development [68]. Nevertheless, recombinant protein vaccines offer several advantages, including a favorable safety profile, defined antigen composition, and the possibility of rational antigen selection, making them attractive candidates for next-generation orthopoxvirus vaccines.

**Figure 2:**
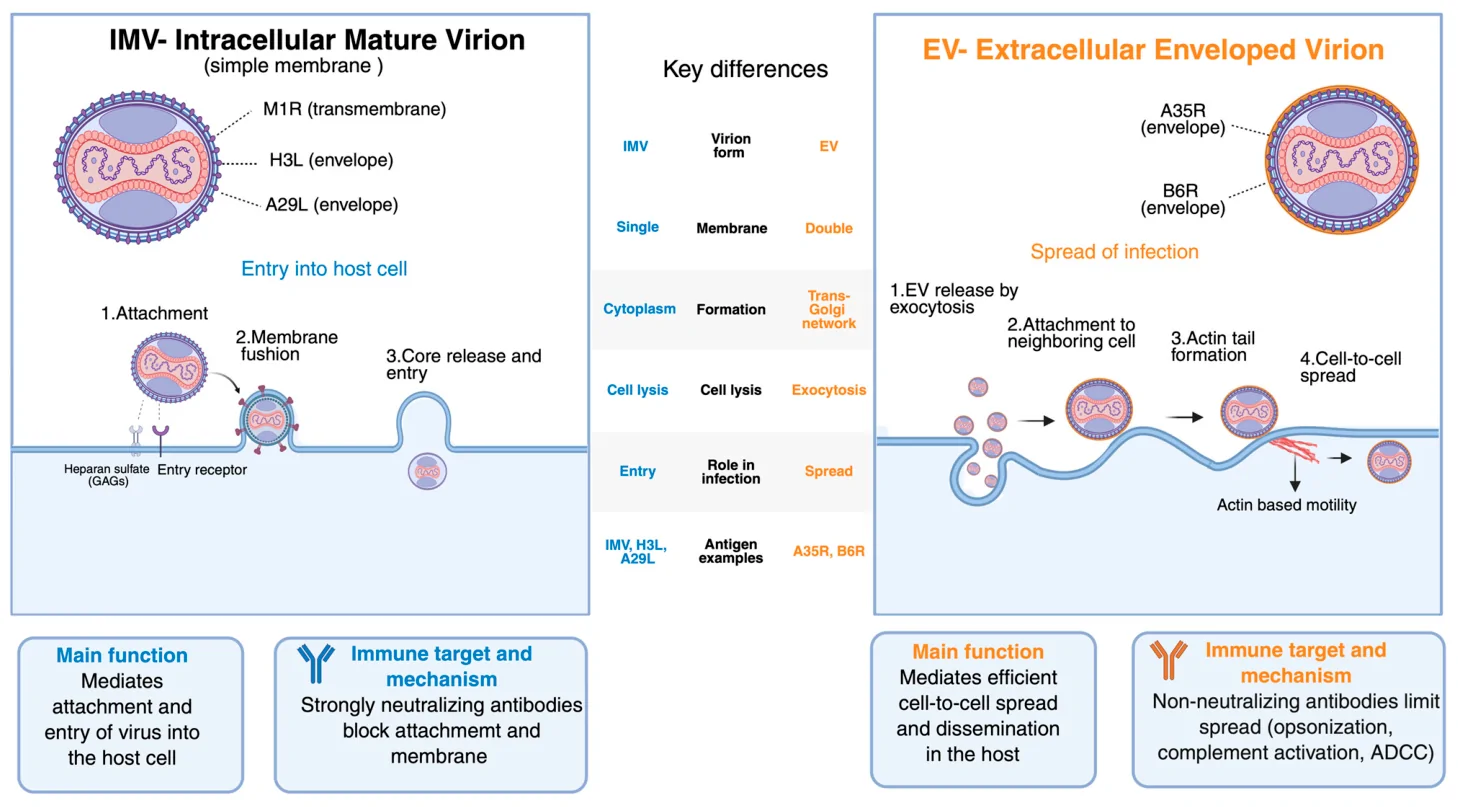
Comparative characteristics of intracellular mature virions (IMVs) and extracellular enveloped virions (EEVs) of Orthopoxviruses and the Role of Their Surface Antigens in Immune Protection. The figure illustrates differences in virion structure, mechanisms of viral entry and dissemination, and the major surface antigens associated with IMV (M1, H3, and A29) and EEV (A35 and B6). Viral entry is mediated by the conserved entry fusion complex (EFC), a multiprotein assembly required for membrane fusion and delivery of the viral core into the host cell. Among EFC-associated proteins, M1 represents one of the principal targets of neutralizing antibodies and contributes substantially to protective immunity against orthopoxviruses.

Recombinant protein vaccines are generally designed according to a common principle whereby the inclusion of antigens derived from different virion forms enables simultaneous targeting of multiple stages of the viral infectious cycle. The core antigen composition typically includes MV-associated proteins, such as the MPXV M1 protein (VACV L1; OPG095), which contribute to neutralization of the mature virion, together with EV-associated antigens, including MPXV A35 (VACV A33; OPG161) and MPXV B6 (VACV B5; OPG190), which are involved in limiting dissemination of the enveloped virion form (Figure 2). Additional antigens, such as MPXV A29 (VACV A27; OPG154) or MPXV E8 (VACV D8; OPG120), may also be incorporated as supplementary targets for neutralizing antibodies [4,5,6,69,70].

Recent cryo-electron microscopy studies have resolved the complete architecture of the orthopoxvirus EFC, demonstrating that it forms a highly organized asymmetric multiprotein assembly that mediates membrane fusion during viral entry. These structural findings provide important insights into the molecular organization of the EFC and the interactions among its components, thereby improving our understanding of the mechanism of orthopoxvirus entry [71].

Subsequent studies consistently demonstrated the effectiveness of combinations of MV- and EV-associated antigens, particularly A35 + B6 + M1. Early studies evaluated recombinant VACV proteins representing both infectious virion forms. Fogg et al. showed that immunization of mice with recombinant VACV A33, B5, and L1 proteins, individually or in combination, induced antibodies directed against EV and MV and protected mice against lethal VACV challenge [4].

Later studies established that both the magnitude and functional activity of these antibody responses directly correlated with protection against lethal viral challenge. In non-human primate models, immunization with recombinant VACV A33, B5, and L1 proteins formulated with the QS-21 adjuvant elicited neutralizing antibodies against MPXV and provided protection against severe disease following MPXV challenge [69]. Xiao et al. further demonstrated that a multicomponent protein-based smallpox vaccine protected mice against vaccinia virus and ectromelia virus challenge [70]. Buchman et al. subsequently showed that a quadrivalent vaccine based on the VACV orthologs A27, A33, B5, and L1 (corresponding to MPXV A29, A35, B6, and M1) protected non-human primates against lethal MPXV challenge [62].

In a study by Berhanu et al., a vaccine formulation based on the VACV orthologs A27, D8, and B5 (corresponding to MPXV A29, E8, and B6) induced high titers of virus-specific antibodies and conferred complete protection against lethal intranasal challenge with VACV WR [6].

Earlier investigations were focused predominantly on classical orthopoxvirus vaccine antigens corresponding to VACV A27, A33, B5, and L1 (MPXV A29, A35, B6, and M1), with particular emphasis on evaluating their protective efficacy in lethal challenge models, including non-human primate studies. More recently, Yang et al. investigated the corresponding MPXV orthologs (A29, M1, A35, and B6) produced in a prokaryotic expression system. These findings extended previous VACV-based studies to the corresponding MPXV proteins and further supported multivalent vaccine strategies specifically tailored to MPXV [72].

Attempts have also been made to minimize antigen composition without compromising immunogenicity. A systematic evaluation of nine multicomponent antigen formulations conducted by Tan et al. demonstrated that a minimal antigen combination consisting of A35 (EEV) and M1 (IMV) was sufficient to induce neutralizing antibody responses comparable to those elicited by more complex multicomponent formulations. The authors further showed that an A35–M1 fusion construct exhibited similar immunogenicity, highlighting the potential for simplifying vaccine formulations without substantial loss of protective efficacy [73].

Current vaccine design strategies increasingly explore recombinant antigens presented in the form of virus-like particles (VLPs) and multivalent nanoparticles. One study demonstrated that multivalent nanoparticles incorporating the M1 (IMV), E8 (IMV), and B6 (EEV) antigens induced high levels of neutralizing antibodies and provided complete protection against lethal VACV challenge in mice [74]. Similarly, a VLP-based subunit vaccine displaying M1 (IMV) together with the EEV-associated antigens A35 and B6 provided multivalent antigen presentation and demonstrated pronounced protective efficacy in preclinical models [75]. Notably, the level of EEV neutralization was shown to correlate more closely with the reduction in viral load than IMV neutralization, further emphasizing the importance of incorporating EEV-associated antigens into subunit vaccine formulations.

Current evidence indicates that the strategic selection of key IMV- and EEV-associated antigens not only enhances the magnitude of protective immune responses but also enables rational optimization of subunit vaccine composition. High protective efficacy has generally been achieved when antigens representing both infectious virion forms are included simultaneously. This “dual form” immunological strategy has been repeatedly demonstrated across experimental models ranging from mice to non-human primates and remains applicable across orthologous antigen systems from VACV to MPXV [5,74,75].

Collectively, current evidence supports the conclusion that rational optimization of antigen composition based on the dual-form immunization concept (IMV + EEV) enables the development of highly effective orthopoxvirus subunit vaccines with improved safety profiles compared with live vaccines. Successful vaccine platforms include the classical A35 + B6 + M1 combination, minimized formulations such as A35 + M1, and innovative nanoparticle-based designs that have demonstrated complete protection against lethal challenge in both preclinical and non-human primate models [4,76].

In addition to their role in vaccine development, recombinant orthopoxvirus proteins are critically important for the design of modern serological diagnostic platforms [76,77,78]. Unlike vaccine development, which primarily aims to induce protective and neutralizing immune responses, serological diagnostics may pursue several distinct objectives, including seroprevalence studies, detection of previous orthopoxvirus exposure, evaluation of vaccine-induced antibody responses, and, where feasible, differentiation between infected and vaccinated individuals [78]. Consequently, the suitability of a recombinant antigen depends not only on its immunogenicity and conservation but also on the intended diagnostic application. Accordingly, different diagnostic objectives require different antigen selection strategies, depending on whether the assay is intended for pan-orthopoxvirus screening, assessment of prior exposure, or differentiation between infected and vaccinated individuals [76,77,78].

A major challenge in the development of serological assays is the high antigenic similarity among orthopoxvirus species, which results in pronounced antibody cross-reactivity. This reflects the extensive conservation of major structural proteins and limits the diagnostic utility of single-antigen approaches. Several recombinant antigens, including H3, A29, B6, A35, and M1, have demonstrated detectable serological reactivity in vaccinated individuals and patients with orthopoxvirus infection, supporting their evaluation as candidate diagnostic antigens. Accordingly, multicomponent antigen panels have been investigated to improve the sensitivity and discriminatory performance of orthopoxvirus serological assays [76,78,79].

Complementary approaches to antigen selection have focused on epitope-level characterization. Because full-length orthopoxvirus proteins contain both diagnostically informative and cross-reactive epitopes, mapping of overlapping peptide regions has been used to identify antigenic determinants with improved diagnostic specificity. These findings demonstrate that epitope characterization complements recombinant protein selection by identifying diagnostically informative regions with reduced cross-reactivity, thereby improving serological assay design [80].

The selection of antigenic targets should take into account the immunobiology of orthopoxviruses and the distribution of major immunodominant antigens across both infectious virion forms. For IMV, the principal neutralizing antigens include MPXV E8, H3, A29, and M1 (corresponding to VACV D8, H3, A27, and L1, respectively). In the case of EEV, the major targets are the transmembrane glycoproteins MPXV B6 and A35 (corresponding to VACV B5 and A33, respectively), which are essential for extracellular enveloped virion morphogenesis, efficient viral dissemination, and cell-to-cell spread [76].

In practical terms, combining multiple immunoreactive orthopoxvirus antigens may broaden antibody detection and improve the diagnostic performance of serological screening assays. In one study, a pooled ELISA comprising MPXV A35, B2, B6, and E8 together with VACV B5 demonstrated higher sensitivity and specificity than the corresponding individual-antigen assays [76].

It should be noted that current reference laboratory guidelines for the confirmation of active mpox infection rely primarily on molecular diagnostic methods, particularly nucleic acid amplification tests (NAATs) and PCR-based detection of viral DNA, whereas serological assays are intended mainly for serosurveillance, retrospective assessment of previous exposure, and evaluation of vaccine-induced immune responses [72]. Serological methods are particularly valuable for seroprevalence studies, confirmation of past infection after viral DNA is no longer detectable, and evaluation of vaccine-induced immunity [76,77,78].

**Figure 3:**
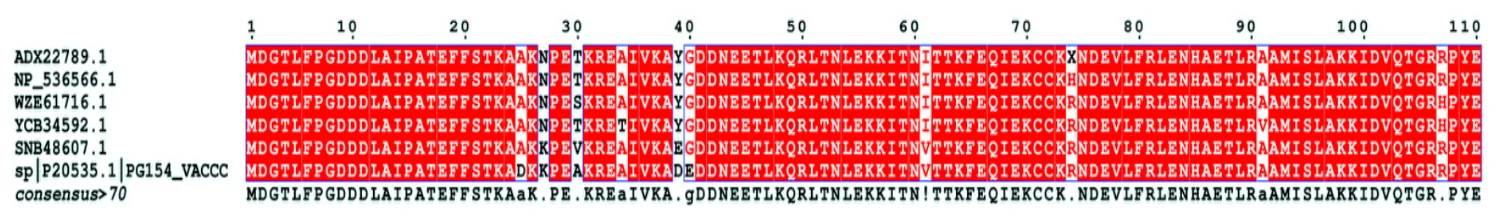
Multiple amino acid sequence alignment of A29 homologues from monkeypox virus (MPXV), cowpox virus (CPXV), and vaccinia virus (VACV). Four representative MPXV sequences were aligned with homologous sequences from CPXV and VACV. Conserved residues are highlighted in red, whereas variable positions indicate amino acid differences among the compared orthopoxvirus homologues. The alignment was generated using Clustal Omega v1.2.4 and formatted for publication using ESPript 3.2.

To further support this comparison, we performed a multiple amino acid sequence alignment of representative MPXV A29 homologues and their orthologues from cowpox virus (CPXV) and vaccinia virus (VACV). The alignment showed that the MPXV sequences were highly conserved, whereas several amino acid substitutions were identified between MPXV and the CPXV/VACV orthologues, which may influence antibody recognition (Figure 3).

The effectiveness of multicomponent antigen strategies has been demonstrated in studies showing that MPXV A29 possesses strong discriminatory capacity for distinguishing mpox-infected individuals from other groups, whereas MPXV M1 performed more effectively as a marker of vaccination with IMVANEX (Modified vaccinia Ankara–based smallpox and mpox vaccine) when analyzed using an optimized assay-specific cutoff, exhibiting moderate sensitivity and high specificity. In the same study, a pooled-antigen ELISA incorporating MPXV A35, B2, B6, and E8 showed high diagnostic sensitivity and specificity. The assay was evaluated using an extensive serum panel comprising negative samples, sera from vaccinated and mpox-infected individuals, and confounder samples from individuals with cytomegalovirus infection, Epstein–Barr virus infection, varicella-zoster virus infection, or rheumatoid factor positivity [76].

Recent studies published between 2023 and 2025 further indicate that recombinant antigen-based panel ELISAs are capable of achieving high diagnostic accuracy for pan-pox antibody screening [76]. In several assay systems, the highest discriminatory performance was associated with the E8 antigen; however, the broader challenge of reliable species-level serological differentiation remains unresolved and requires further investigation [78].

For diagnostic applications, the use of multicomponent antigen panels incorporating both IMV- and EEV-associated antigens remains essential for reliable species differentiation and seroprevalence screening. Nevertheless, distinguishing between previously infected and vaccinated individuals remains a major challenge because of extensive serological cross-reactivity. In addition to integrated molecular-serological diagnostic approaches, recent studies have demonstrated that evaluating the antibody binding ratio to homologous MPXV and VACV antigens, particularly the homologous MPXV A35 and VACV A33 proteins, provides an additional parameter for distinguishing mpox infection from vaccinia vaccination and may complement multicomponent antigen panel-based serological assays [76,79].

In the development of both recombinant protein-based vaccines and serological diagnostic platforms for orthopoxviruses, primary consideration should be given to the functional differences between the two infectious virion forms and their respective roles in antiviral immunity and immunodiagnostics [74,81].

## 4. Criteria for the Selection of Recombinant Antigens for Orthopoxvirus Diagnostics

The selection of recombinant antigens largely determines the sensitivity and specificity of serological diagnostic systems. In the case of *Orthopoxvirus* species, this task is complicated by the high degree of antigenic similarity among members of the genus, as antibodies generated against one orthopoxvirus frequently recognize proteins from other related species, resulting in pronounced cross-reactivity [81]. Consequently, antigen selection should be based not only on immunogenicity but also on the combined evaluation of biological and technological characteristics.

Proteome-wide serological profiling demonstrated that orthopoxvirus infection elicits antibody responses against both surface-associated and internal viral proteins [82]. Accordingly, antibody responses are not limited to surface proteins but may also be directed against internal and non-structural viral antigens. Although such antigens are generally less relevant as targets of neutralizing antibodies, some have demonstrated diagnostic value, particularly for differentiating infection-induced and vaccine-induced immune responses [76,83]. These findings indicate that recombinant antigen selection should consider not only protein localization but also antigen immunodominance and the frequency of serological recognition [82]. Furthermore, the marked inter-individual heterogeneity of antibody responses further supports the development of multiplex antigen panels for serological assays.

Immunogenicity is another critical determinant influencing assay sensitivity. Proteins capable of inducing stable and reproducible antibody responses in the majority of infected or vaccinated individuals are considered the most suitable antigenic targets [74,81]. By contrast, weakly immunogenic proteins may contribute to underestimation of seropositive samples.

Sequence conservation has a dual diagnostic significance. Highly conserved proteins are advantageous for pan-orthopoxvirus screening but simultaneously increase the risk of serological cross-reactivity. Conversely, variable epitopes may provide greater value for differential diagnostics. Therefore, the optimal selection of recombinant antigens ultimately depends on the intended diagnostic application.

Antigen specificity is inherently limited by the high degree of similarity among orthopoxvirus proteins, and therefore the use of a single antigen rarely provides sufficient diagnostic accuracy [74,76,77,81]. In this context, multicomponent antigen approaches are generally more effective, as they reduce the proportion of false-positive results and improve the reliability of result interpretation.

Preservation of antigen structure is also critical for proper epitope recognition. Disruption of protein conformation during expression or purification may substantially reduce immunoreactivity [84], even when the amino acid sequence itself remains unchanged [85].

From a practical perspective, technological suitability is equally important. Recombinant antigens should demonstrate stable expression, adequate solubility, and reproducible purification characteristics. In addition, the choice of expression system may influence the structural and immunological properties of recombinant proteins, since different platforms vary in their ability to perform post-translational modifications, including glycosylation, disulfide bond formation, and proper protein folding. Such differences can affect epitope presentation and antibody recognition, particularly for glycoproteins associated with the extracellular enveloped virion [86]. These properties are essential for the standardization and large-scale implementation of diagnostic platforms [87,88].

An additional consideration is the existence of the two infectious virion forms, IMV and EV, which differ in their surface protein composition. The inclusion of antigens derived from both virion forms enables more comprehensive detection of antibody responses and improves assay sensitivity [89,90,91].

Taken together, the optimal selection of recombinant antigens should be based on the combined evaluation of antigen accessibility, immunogenicity, diagnostically relevant variability, structural stability, and technological suitability. In most cases, the best diagnostic performance is achieved using multicomponent antigen panels [79,92,93,94].

Several orthopoxvirus antigens, including A29, H3, E8, M1, A35, and B6, have been identified as important immunogenic targets and have been extensively investigated for serological assays and vaccine development [76,83,92]. In addition, differential recognition of orthologous MPXV and VACV antigens (MPXV A35 versus VACV A33, MPXV B6 versus VACV B5, and MPXV E8 versus VACV D8) has been reported and may facilitate discrimination between sera from vaccinated and MPXV-infected individuals [76,92].

## 5. Major Recombinant Orthopoxvirus Proteins: Structure and Function

Recent studies have focused extensively on individual orthopoxvirus proteins as potential antigenic targets for serological diagnostics and vaccine development. Particular attention has been directed toward structural proteins localized on the surface of different infectious virion forms, as these proteins represent the primary targets of humoral immune responses. Among the most extensively investigated antigens are A29, M1, H3, E8, A35, and B6, all of which exhibit pronounced immunogenicity and are actively studied as components of multicomponent diagnostic and vaccine platforms [3,4,5,6,69,70,74,75].

The principal structural, functional, and immunological characteristics of major Orthopoxvirus recombinant proteins, including their molecular weights, biological roles, and diagnostic and vaccine potential, are summarized in Table 3 based on published experimental data.

Among the proteins discussed, A35, B6, H3, M1, and A29 were selected for further analysis because of their potential utility in the development of diagnostic and vaccine platforms. Their selection was based on their critical roles in the viral life cycle, high immunological relevance, and demonstrated performance in experimental vaccine and serological studies. Collectively, these antigens represent both the intracellular mature virion (IMV) and extracellular enveloped virion (EV) forms and participate in key processes involved in viral entry, dissemination, and immune recognition. A detailed analysis of their structural, functional, and immunological properties provides a biological basis for understanding their suitability as candidate antigens for diagnostic and vaccine applications.

### 5.1. MPXV A35 Protein

From a functional perspective, A35 is considered one of the principal antigens associated with the extracellular enveloped virion (EEV) form. The protein is localized on the outer membrane of extracellular enveloped virions (EV; IEV/CEV/EEV) and represents an envelope glycoprotein orthologous to the vaccinia virus A33 protein [66,72,137] A35 participates in the structural organization of EEV particles and contributes to cell-to-cell viral spread, underscoring its importance in the orthopoxvirus infectious cycle [72,138].

The immunological properties of A35 make it one of the most promising targets for the development of vaccines and diagnostic systems. A35 is regarded as an immunodominant MPXV antigen, with strong antibody responses frequently reported against both A35 and its VACV homolog A33. More broadly, EEV-associated surface proteins, including A33/A35, demonstrate particularly strong immunodominance among orthopoxvirus antigens [138].

A35 efficiently induces the production of virus-neutralizing antibodies with demonstrated protective potential [72]. This activity is associated with the presence of accessible neutralizing epitopes on the EEV membrane, enabling efficient recognition by the host immune system [67]. Importantly, the immunogenicity of A35 depends strongly on preservation of its native conformational structure. Disruption of the native protein conformation is accompanied by reduced humoral immune responses despite the retention of T-cell reactivity. In contrast, conformationally preserved A35 induces robust antibody responses, whereas the isolated soluble form exhibits substantially lower immunogenicity.

The immune response elicited by A35 is multifaceted and includes both humoral and cellular components. The protein is capable of inducing antigen-specific T-cell responses, including activation of CD4+ T lymphocytes; however, the presence of cellular immunity does not always directly correlate with protective efficacy, highlighting the predominant role of neutralizing antibodies in A35-mediated protection [72].

Experimental studies have shown that multicomponent formulations combining IMV- and EEV-associated antigens provide superior protective efficacy compared with individual immunogens [137].

In addition to its vaccine potential, A35 is considered a promising marker for serological diagnostics. Together with the homologous H3 protein, A35 has been identified as both a B-cell and serological marker of MPXV infection. These properties support its application in immunoassay-based diagnostic platforms, including ELISA systems, for the detection of recent infection [139].

For comparison, the VACV ortholog A33 is an EEV-associated envelope glycoprotein that contributes to extracellular enveloped virion (EEV) morphogenesis and viral dissemination [90]. Although A33 is not required for recruitment of these proteins to wrapping membranes, its absence disrupts the completion of intracellular enveloped virion (IEV) morphogenesis, abolishes the formation of actin-containing microvilli, and reduces the efficiency of cell-to-cell viral spread [140].

Taken together, A35 functions primarily as an immunogenic extracellular virion antigen that contributes to viral dissemination and protective immune recognition. In contrast, A33 performs predominantly structural and functional roles related to the formation of enveloped virions and efficient viral dissemination.

### 5.2. MPXV B6 Protein

The B6 protein belongs to the antigenic repertoire of the extracellular enveloped virion (EV/EEV) form of orthopoxviruses and is characterized by high antigenic accessibility. It is a palmitoylated envelope glycoprotein localized on the surface of extracellular viral particles and represents the ortholog of the vaccinia virus B5 protein [72,141].

Functionally, the VACV B5 protein, the ortholog of MPXV B6, is required for intracellular mature virion wrapping, extracellular enveloped virion formation, and efficient viral dissemination [142].

Immunological studies, including analyses based on phage display technologies, have demonstrated the ability of B6 to bind a broad spectrum of fully human monoclonal antibodies, indicating the exposed nature and pronounced immunodominance of its epitopes [143]. The protein contains multiple spatially distinct antigenic determinants recognized by different antibody populations, reflecting substantial epitope complexity. Neutralizing epitopes are formed predominantly by conserved amino acid residues, although individual amino acid substitutions may reduce binding affinity without completely abolishing antibody recognition, illustrating the fine balance underlying cross-reactive immune responses [74].

The high degree of conservation among B6 orthologs across orthopoxvirus species enables cross-recognition of MPXV, VACV, VARV, and CPXV proteins by human monoclonal antibodies [74]. This property makes B6 a particularly attractive target for the induction of cross-protective humoral immunity and highlights its importance in the development of pan-orthopoxvirus vaccines and diagnostic platforms.

Antibodies directed against B6 efficiently neutralize extracellular enveloped virions (EV) of mpox virus. Comparative studies have shown that anti-B6 antibodies exhibit stronger binding and neutralizing activity against EV particles than antibodies directed against M1 [141]. At the same time, orthopoxvirus neutralization is complementary in nature: anti-M1 antibodies predominantly target the MV/IMV form, whereas anti-B6 antibodies are primarily active against EV particles. This reflects the existence of distinct vulnerable antigenic targets associated with different infectious virion forms and provides a strong rationale for incorporating both antigens into multicomponent vaccine and diagnostic formulations [141].

Antibodies targeting B5, the VACV ortholog of MPXV B6, are major contributors to the neutralization of EEV particles [138]. In addition, B6 predominantly induces humoral immunity directed against extracellular virions while also being capable of eliciting moderate cellular immune responses [144].

Additional evidence indicates that the immunogenicity of B6 can be further enhanced through the use of modern adjuvant systems. For example, a combination of B6 with the BC02 adjuvant induces Th1-oriented cellular immunity together with durable effector and memory B-cell responses [144]. In animal models, mice immunized with B6-containing vaccine formulations were protected against lethal MPXV challenge, while vaccine-induced neutralizing antibodies efficiently suppressed viral replication and reduced pathological tissue damage [74].

Collectively, B6 represents a key antigen of the extracellular enveloped orthopoxvirus form, characterized by high sequence conservation, pronounced epitope complexity, and an essential functional role in viral dissemination. Its ability to induce potent neutralizing humoral responses and promote cross-protective immunity makes B6 one of the most promising components of multivalent orthopoxvirus vaccines and serological diagnostic systems.

### 5.3. MPXV H3 Protein

The H3 protein is one of the major surface components of the intracellular mature virion (IMV) form of orthopoxviruses and exhibits a high degree of sequence conservation, reflecting its important functional role in the viral life cycle [145]. H3 is a 324-amino-acid transmembrane protein (p35) localized on the IMV surface and encoded by the *H3L* gene [108,109]. Owing to its structural organization, H3 plays a central role during the early stages of infection, including viral adsorption to host cells and subsequent viral entry.

Functionally, H3 participates in the initial stages of virus–cell interaction through cooperation with other adhesion proteins and components of the membrane fusion complex. Its α-helical domain mediates specific binding to cell-surface heparan sulfate and functions as a receptor-binding module [110,145]. Soluble H3 is capable of competing for heparan sulfate binding and thereby blocking IMV adsorption, further confirming its essential role in viral attachment. In addition, H3 deletion mutants display a small-plaque phenotype and reduced viral production, indicating that the protein contributes not only to viral adsorption but also to virion morphogenesis [146].

From structural and evolutionary perspectives, H3 is characterized by high amino acid conservation across different orthopoxviruses, including MPXV and VACV (approximately 93.5% sequence identity), which underlies its pronounced cross-antigenicity [145]. Homologs of H3 have been identified not only within the genus *Orthopoxvirus* but also in other members of the family *Poxviridae*, indicating evolutionary conservation of both structure and function [108,109]. Antibodies directed against H3 following infection with different orthopoxviruses exhibit broad cross-recognition, including reactivity with Orf virus, capripoxviruses, and fowlpox virus antigens [108].

The immunological properties of H3 indicate pronounced immunodominance. The protein is consistently recognized by high-titer antibodies in most individuals, particularly following booster immunization. Protein microarray studies have identified H3 as one of the major targets of humoral immune responses induced by smallpox vaccination. H3 also induces strong immune responses in both animals and humans [108].

Mapping of antigenic determinants has identified at least four spatially distinct linear epitopes, indicating considerable epitope modularity and antigenic complexity [132]. Such structural organization contributes to the development of multifocal immune responses and increases the likelihood of recognition by diverse antibody populations.

Antibodies directed against H3 exhibit both direct virus-neutralizing activity and complement-dependent antiviral effects. Purified human anti-H3 antibodies demonstrate significant virus-neutralizing activity in vitro. Immunization with recombinant H3 induces high titers of neutralizing antibodies and protects animals against lethal vaccinia virus challenge. Similarly, passive transfer of anti-H3 serum confers protection against viral infection [108].

Despite its high immunogenicity, the neutralizing activity of H3 alone may be less pronounced than that of some other orthopoxvirus antigens, supporting its optimal use as part of multicomponent vaccine formulations. The surface exposure of H3 and the sensitivity of specific domains to mutational changes also make the protein a promising target for antiviral strategies aimed at blocking early stages of infection [145].

In addition to its vaccine relevance, H3 possesses considerable diagnostic value. Together with A35, H3 has been identified as a serological and B-cell marker of MPXV infection and may be incorporated into ELISA-based diagnostic platforms for the detection of recent infection [139]. H3 also demonstrates stable recognition in immunoblot analyses, where antigens with molecular masses of 30–35 kDa, including H3, are consistently detected in sera from immunized animals and humans [146].

Collectively, H3 represents a highly conserved and immunodominant antigen of the IMV form of orthopoxviruses, involved in viral adsorption, virion morphogenesis, and induction of protective immune responses. Its combination with EV-associated antigens such as B6 and A35 enables broader coverage of distinct stages of the viral life cycle and enhances the overall effectiveness of vaccine and diagnostic platforms.

### 5.4. MPXV M1 Protein

The M1 protein is a surface-associated component of the intracellular mature virion (IMV) form of monkeypox virus and plays a critical role in virion maturation and viral entry into host cells. M1 is a highly conserved myristoylated membrane protein of the IMV surface and represents the ortholog of the vaccinia virus L1 protein [67,95].

Localized on the IMV surface, M1 contributes to virion formation and morphogenesis, making it essential for the maintenance of viral infectivity [86,147]. Functional studies have demonstrated that M1 is directly involved in virion assembly, and disruption of its activity results in defective morphogenesis and reduced infectious viral titers. In addition, M1 participates in viral entry and therefore represents one of the principal targets for blocking the early stages of orthopoxvirus infection [95,138]. This role is further supported by studies demonstrating the neutralizing activity of antibodies and nanobodies directed against M1, which are capable of inhibiting viral infection [138].

Structurally, M1 contains several spatially distinct epitopes capable of inducing neutralizing antibody responses [143]. It is considered one of the most conserved orthopoxvirus proteins, which contributes to its pronounced cross-antigenicity and supports its utility as a broadly applicable target for vaccine development. In particular, the orthologous L1 protein, together with A29, represents one of the major targets of neutralizing antibodies against orthopoxviruses [66,96,97].

Neutralizing monoclonal antibodies targeting M1 have demonstrated potent antiviral activity and significant protective efficacy in vivo, highlighting M1 as an important target of protective humoral immunity [141].

Despite its considerable efficacy as an individual immunogen, the greatest protective benefit is achieved when M1 is incorporated into multicomponent antigen formulations. Multicomponent vaccine formulations incorporating both IMV- and EEV-associated antigens provide broader and more effective protection than individual antigens administered alone [67,137]. Such combinations have demonstrated protective efficacy in both lethal orthopoxvirus challenge models and a non-lethal MPXV challenge model [66,67,74,147].

The practical relevance of M1 is further supported by its incorporation into multiple modern vaccine platforms. The protein has been successfully utilized in mRNA vaccines, recombinant subunit vaccines, and DNA vaccine formulations [66,67,85]. In particular, vaccine formulations containing combined M1 and A35 antigens induce stronger immune responses involving both humoral and cellular components [85].

Collectively, M1 represents a key highly conserved antigen of the IMV form of orthopoxviruses that participates in virion assembly, morphogenesis, and viral entry. Its pronounced immunogenicity, ability to induce potent neutralizing antibodies, and synergistic effects when combined with EEV-associated antigens make M1 one of the central components of contemporary multivalent vaccine and diagnostic strategies.

### 5.5. MPXV A29 Protein

The A29 protein is a surface antigen of the intracellular mature virion (IMV) form of monkeypox virus and plays an important role during the early stages of virus–host cell interaction. A29 is a membrane-associated surface protein and the ortholog of the vaccinia virus A27 protein, sharing a high degree of amino acid homology (approximately 95%), which reflects its evolutionary conservation. Localized on the IMV surface, A29 participates in viral adhesion to host cells by mediating primary attachment through interactions with glycosaminoglycans, including heparan sulfate [148].

Functionally, A29 is directly involved in viral attachment to the cell surface. The protein interacts with heparin and heparan sulfate, thereby facilitating IMV adsorption and promoting subsequent viral entry [72,146,149]. The VACV A27 protein, the ortholog of MPXV A29, mediates viral attachment through interaction with cell-surface heparan sulfate [117,148]. Viral adhesion is additionally mediated by other attachment proteins, including H3, which binds heparan sulfate, and D8 (MPXV E8), which binds chondroitin sulfate, indicating functional overlap among orthopoxvirus attachment mechanisms [107,112,117]. Beyond its role in viral adhesion, A29 and its VACV ortholog A27 are implicated in later stages of the viral life cycle. Functional disruption of VACV A27 results in defective MV transport and egress, impaired EV formation, and a small-plaque phenotype, highlighting its importance in efficient viral dissemination [120].

From an immunological perspective, A29 exhibits pronounced immunogenicity and is capable of inducing antigen-specific humoral immune responses. However, despite generating highly specific antibodies, anti-A29 antibodies display limited neutralizing activity and do not efficiently block infection [148]. These findings indicate that A29 functions primarily as an accessory adhesion factor rather than as a dominant neutralizing target, in contrast to several other orthopoxvirus antigens.

A29 shares a high degree of sequence homology with the VACV ortholog A27, supporting its potential as a cross-reactive orthopoxvirus antigen while retaining MPXV-specific diagnostic features [148]. At the same time, the presence of several variable amino acid residues, including positions 27, 30, 39, and 40, enables discrimination between MPXV and VACV proteins at the monoclonal antibody level. In particular, the monoclonal antibody 2–5H has been shown to exhibit high specificity for MPXV A29 without cross-reactivity toward VACV A27, highlighting the diagnostic relevance of this antigen [148].

The vaccine potential of A29 is realized primarily within multicomponent antigen formulations. Although the protein demonstrates limited protective efficacy as an individual immunogen, its incorporation into antigen combinations substantially enhances immune responses. In particular, DNA vaccines expressing combinations of IMV- and EEV-associated antigens, including A29, A35, M1, and B6, provide protection against lethal challenge with multiple orthopoxviruses, including mpox virus, vaccinia virus, and rabbitpox virus [66].

Collectively, A29 represents a conserved surface protein of the IMV form that plays important roles in viral adhesion and morphogenesis. Despite the limited neutralizing activity of anti-A29 antibodies, its involvement in early stages of infection and its pronounced immunogenicity make A29 a valuable component of multivalent vaccine platforms and a promising marker for MPXV-specific diagnostics.

## 6. Phylogenetic Analysis of Key Orthopoxvirus Genes

**Figure 4:**
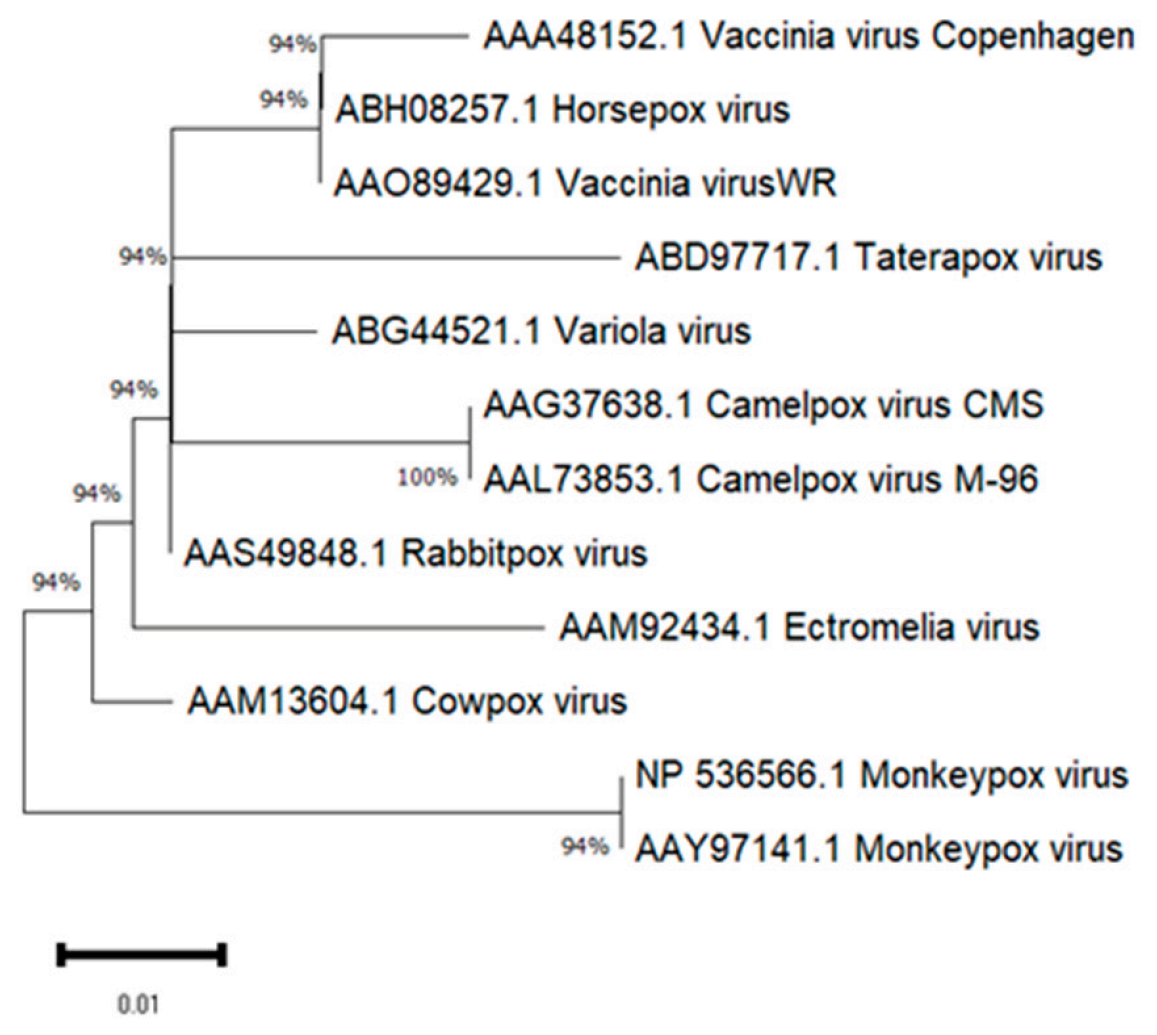
Phylogenetic Tree of A29 Amino Acid Sequences among *Orthopoxvirus* Species.

**Figure 5:**
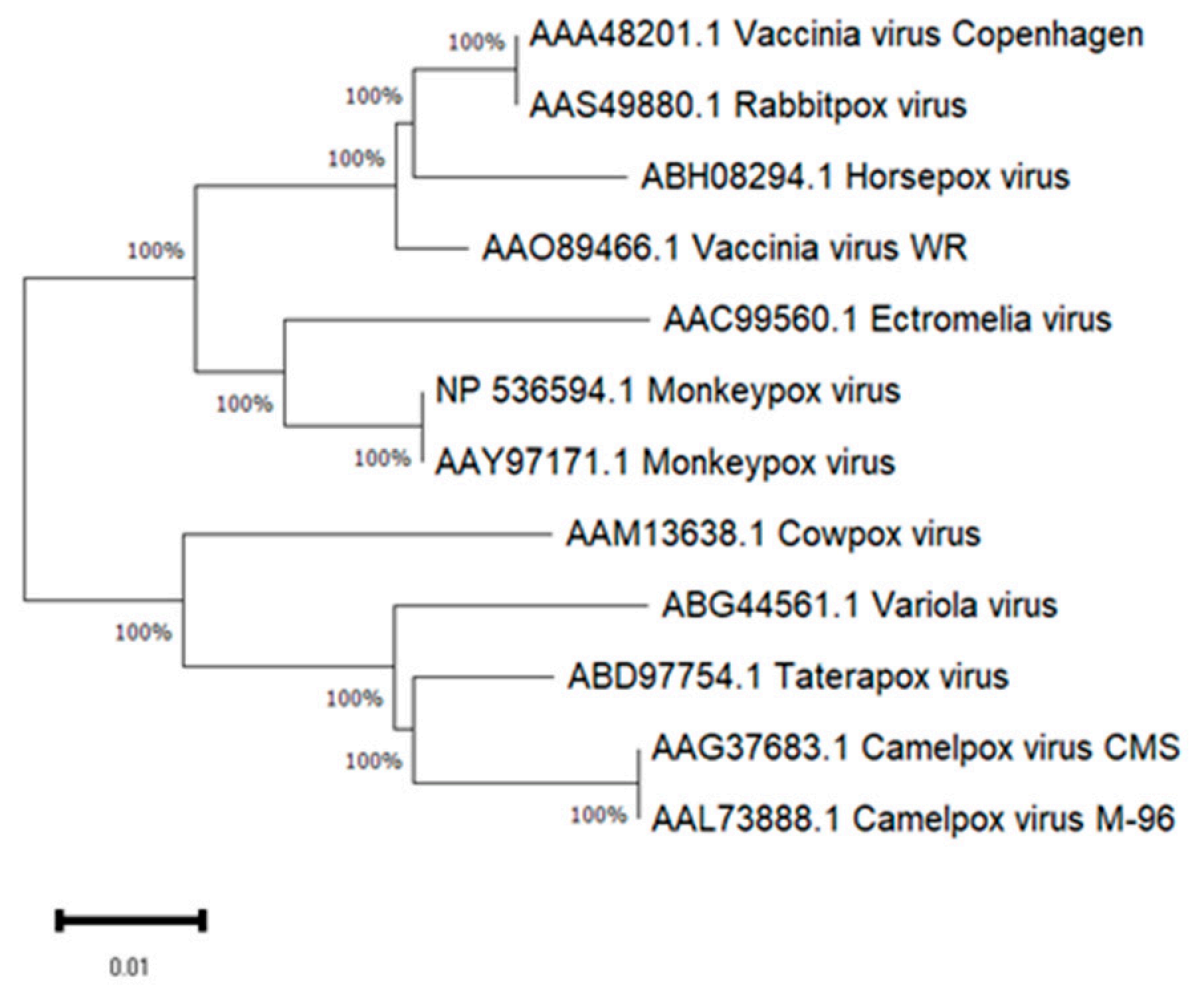
Phylogenetic Tree of B6 Amino Acid Sequences among *Orthopoxvirus* Species.

**Figure 6:**
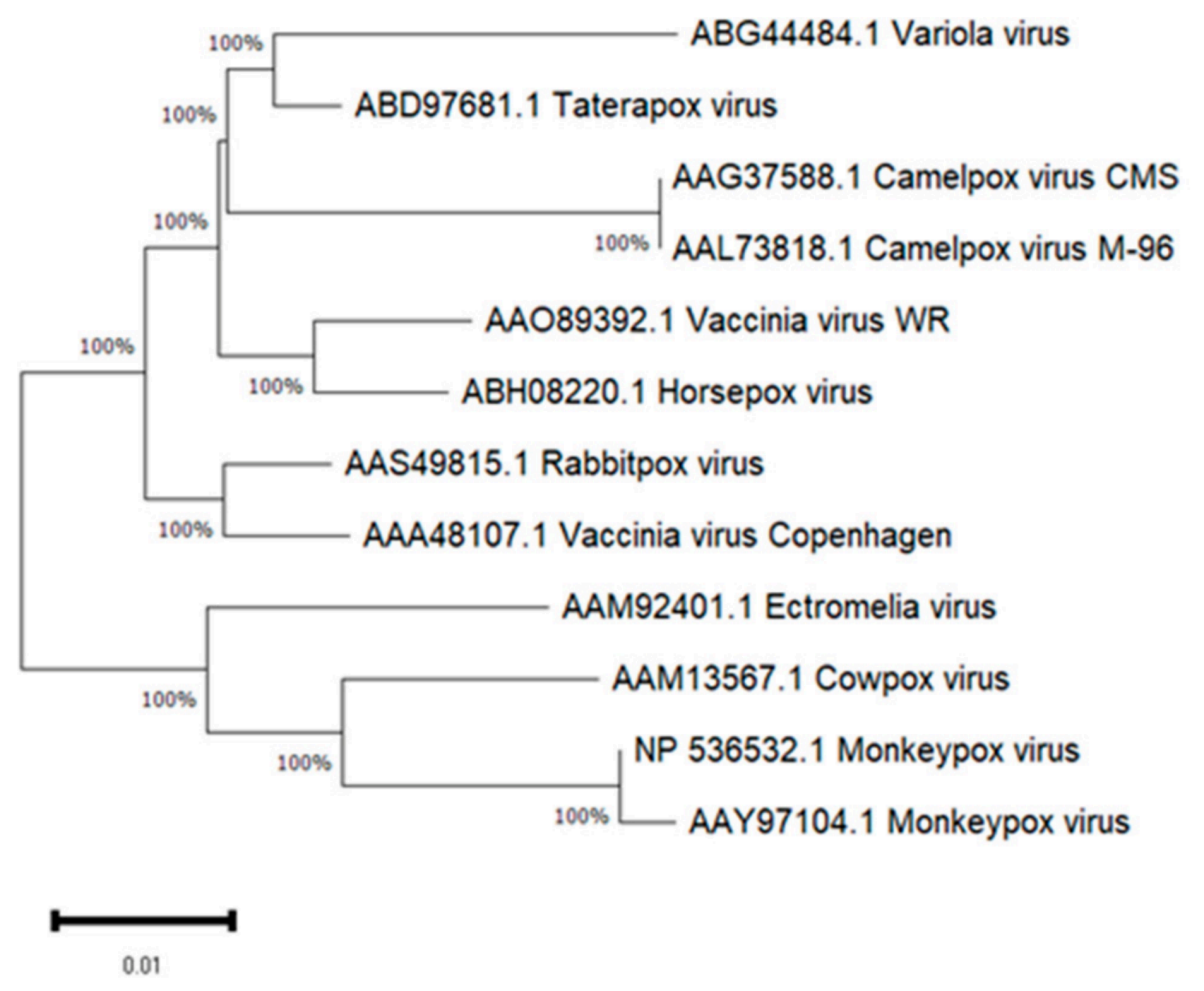
Phylogenetic Tree of E8 Amino Acid Sequences among *Orthopoxvirus* Species.

**Figure 7:**
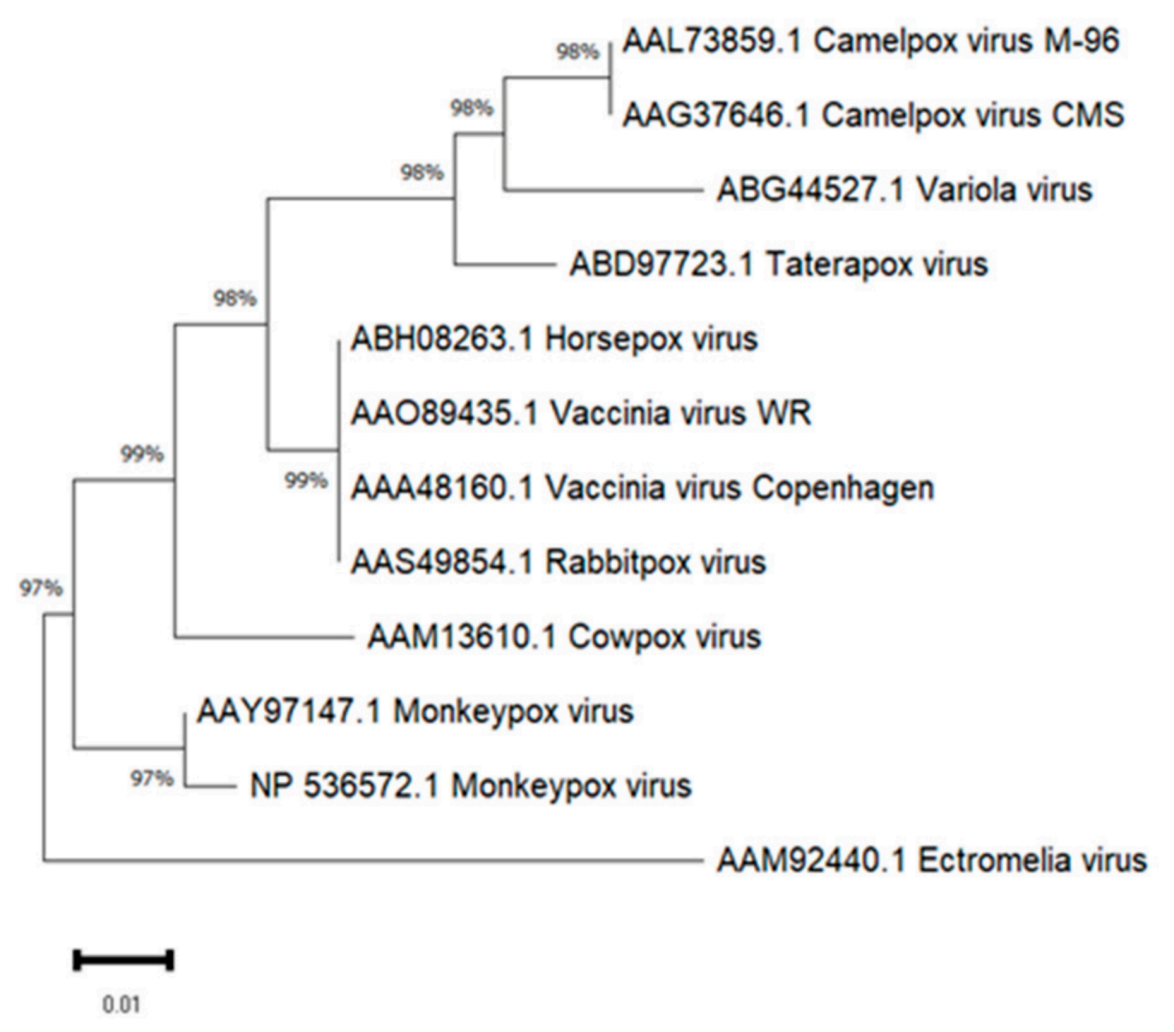
Phylogenetic Tree of A35 Amino Acid Sequences among *Orthopoxvirus* Species.

**Figure 8:**
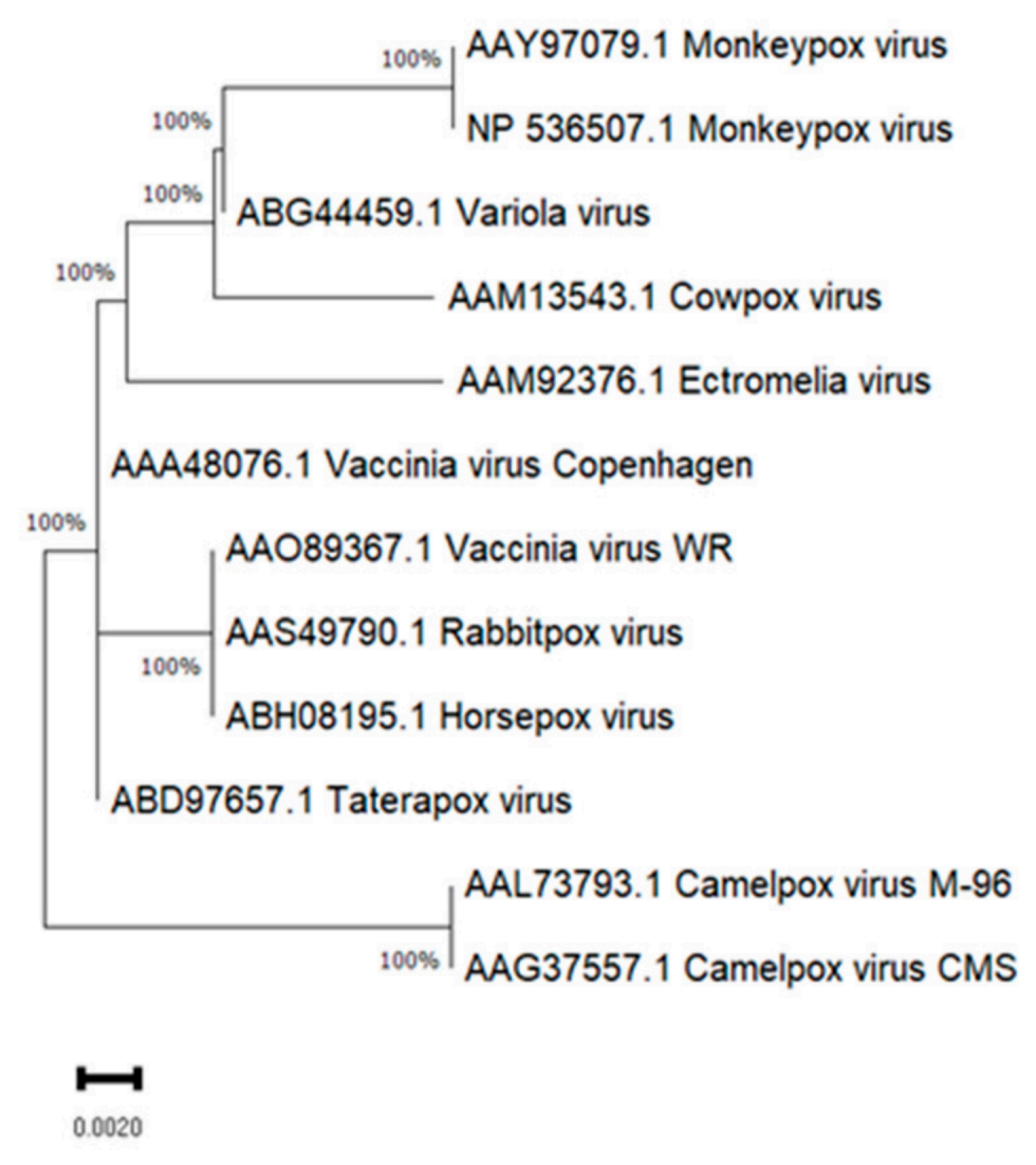
Phylogenetic Tree of M1 Amino Acid Sequences among *Orthopoxvirus* Species.

Phylogenetic analysis of five structurally and functionally important genes (A29L, B6R, E8L, A35R, and M1R) demonstrated a high degree of sequence conservation among orthopoxviruses, reflected by the short branch lengths observed in the phylogenetic trees (0.002–0.01 substitutions per site). Although the overall tree topologies were largely similar, differences in the clustering patterns of individual strains were observed depending on the gene analyzed (Figure 4, Figure 5, Figure 6, Figure 7 and Figure 8).

Phylogenetic analysis of the A29 amino acid sequences revealed two major phylogenetic lineages with several well-supported internal clades (Figure 4). One lineage comprised vaccinia virus, horsepox virus, variola virus, camelpox virus, and taterapox virus, whereas the second included rabbitpox virus, ectromelia virus, cowpox virus, and monkeypox virus. Within the latter lineage, the two MPXV isolates formed a strongly supported monophyletic clade, with cowpox virus representing the closest sister lineage. Vaccinia Copenhagen and horsepox virus also formed a closely related cluster, while the two camelpox virus isolates grouped together with strong bootstrap support.

Phylogenetic analysis of the B6 amino acid sequences revealed a phylogenetic topology that differed from that observed for A29. Members of the genus were separated into two major monophyletic groups. The first group included vaccinia virus, rabbitpox virus, horsepox virus, ectromelia virus, and monkeypox virus, whereas the second comprised cowpox virus, variola virus, taterapox virus, and camelpox virus (Figure 5). Notably, ectromelia virus clustered most closely with monkeypox virus within this lineage, indicating a high degree of sequence similarity in the B6 protein.

Analysis of the E8 amino acid sequences revealed a similar two-cluster phylogenetic topology. The first group included vaccinia virus, rabbitpox virus, horsepox virus, taterapox virus, variola virus, and camelpox virus, whereas the second comprised cowpox virus, ectromelia virus, and monkeypox virus (Figure 6). Consistent with the B6 phylogeny, the two MPXV isolates formed a well-supported clade, with cowpox virus representing the closest sister lineage.

Phylogenetic analysis of the A35 amino acid sequences revealed the most complex phylogenetic topology, consisting of five principal lineages (Figure 7). One well-supported lineage comprised taterapox virus together with variola virus and camelpox virus (BS = 98%). A second lineage included vaccinia virus copenhagen, vaccinia virus WR, horsepox virus, and rabbitpox virus (BS = 99%), indicating a high degree of sequence similarity among these viruses. cowpox virus, the two monkeypox virus isolates, and ectromelia virus each occupied distinct phylogenetic positions within the tree.

Analysis of the M1 amino acid sequences revealed a phylogenetic topology distinct from that of A35, comprising several well-supported lineages (Figure 8). The two MPXV isolates formed a strongly supported clade that clustered successively with variola virus and cowpox virus. vaccinia virus Copenhagen, vaccinia virus WR, rabbitpox virus, and horsepox virus formed a closely related lineage, whereas ectromelia virus, taterapox virus, and the camelpox virus clade occupied separate phylogenetic positions within the tree.

The phylogenetic analysis revealed a complex pattern of evolutionary relationships within the genus *Orthopoxvirus*. Although the overall tree topologies differed among the analyzed proteins, the A29, B6, and E8 trees showed relatively simple clustering patterns, whereas the A35 and M1 trees displayed more complex branching topologies with multiple well-supported lineages. The low overall branch lengths (0.002–0.01 substitutions per site) indicate a high degree of sequence conservation among the analyzed proteins, consistent with their essential structural and functional roles and strong evolutionary constraints.

The clustering patterns observed in the present study are generally consistent with previously published phylogenetic analyses, which likewise demonstrate close evolutionary relationships among several orthopoxvirus species, particularly variola virus, camelpox virus, and taterapox virus, while also supporting the close relatedness of monkeypox virus and cowpox virus. Ectromelia virus consistently occupied a distinct phylogenetic position across the analyzed datasets [16,17,30].

These evolutionary relationships provide a biological framework for interpreting antigen conservation and the extensive serological cross-reactivity observed among closely related orthopoxviruses. At the same time, differences in phylogenetic clustering among individual genes suggest that conserved proteins may differ in their discriminatory potential and should therefore be considered during the selection of recombinant antigens for multicomponent serological assays aimed at improving species differentiation.

## 7. Discussion

Analysis of the published data indicates that the selection of recombinant proteins for the diagnosis and prevention of orthopoxvirus infections cannot be based solely on immunogenicity or the degree of sequence conservation. Equally important is understanding the specific role of each protein in the viral life cycle, its localization, and its accessibility to antibodies. These considerations likely explain why current research increasingly focuses not on individual antigens but rather on rational antigen combinations incorporating proteins derived from both infectious virion forms, IMV and EV [51,58].

This approach appears particularly well justified when the functional differences between these virion forms are considered. IMV-associated proteins, especially M1 and H3, are primarily involved in viral attachment and entry into host cells, whereas EV-associated proteins, including A35 and B6, contribute mainly to viral dissemination between cells [51,58]. Consequently, the immune responses induced by these antigens cannot be regarded as functionally interchangeable. Antibodies directed against IMV antigens play a more prominent role in direct viral neutralization, whereas antibodies targeting EV antigens are more important for restricting subsequent viral spread. This interpretation is consistent with findings from preclinical studies in which the strongest protective effects were achieved through combined immunization with antigens from both virion forms rather than with single-antigen formulations [59,60,61,66].

At the same time, the high antigenic similarity among orthopoxviruses remains a double-edged characteristic. On the one hand, it enables the development of group-specific serological assays. On the other hand, it complicates interpretation of serological results, particularly when differentiation is required between infection caused by a heterologous orthopoxvirus, such as MPXV, and immune responses induced by prior vaccination or exposure to other orthopoxviruses [69,70,71,72]. In this context, reliance on a single antigen appears insufficient. Even highly immunodominant proteins do not always provide the level of diagnostic accuracy required when their epitopes are recognized in a cross-reactive manner. For this reason, multicomponent antigen panels incorporating both conserved and more discriminative antigenic targets appear to represent a more promising strategy [70].

From a practical standpoint, M1 and B6 are of particular interest. M1 remains one of the most potent IMV-associated targets because of its ability to induce strong humoral immune responses and neutralizing antibodies, making it attractive for both vaccine formulations and serological diagnostic systems [69,150]. In contrast, B6 is especially important as an EV-associated antigen. Its significance is determined not only by its immunogenicity but also by the observation that immune responses directed against B6 correlate with restriction of viral dissemination in vivo. This makes B6 a particularly valuable component of multicomponent antigen formulations intended to target different stages of the infectious process [67,138,147].

A29 occupies a somewhat different position. This protein is of interest as an IMV surface antigen and may be particularly useful for diagnostic applications, especially in the detection of antibodies directed against MPXV-associated targets. However, available data suggest that its role in the induction of highly effective protective immunity is more limited. In other words, A29 appears to function more effectively as a diagnostic marker than as a standalone component for vaccine design [132,143,145]. Against this background, H3 retains importance as a conserved surface antigen that may be suitable for inclusion in serological panels, particularly when increased assay sensitivity is required [99,100,101,102,103,132,139].

Particular attention should also be given to A35. This protein is noteworthy not only as an EV-associated antigen but also as a factor linked to virulence and virus–host immune interactions. Structural preservation is especially critical for A35. Disruption of its native conformation may substantially weaken humoral immune responses, even when certain antigenic determinants remain preserved [130]. This observation has important practical implications: the diagnostic and immunogenic value of recombinant proteins depends not only on their intrinsic biological properties but also on whether they are correctly produced and retain the necessary conformational epitopes following expression and purification.

The phylogenetic analyses presented in this study generally confirm the high degree of conservation of the selected proteins among members of the genus *Orthopoxvirus*. On the one hand, these findings support the potential use of such antigens in pan-orthopoxvirus diagnostic systems. On the other hand, even relatively minor interspecies differences may influence antigenic structure and, consequently, antibody-binding characteristics. Therefore, phylogenetic relatedness alone does not guarantee equivalent diagnostic performance, and each candidate antigen requires independent experimental validation using well-characterized serum panels.

It should be acknowledged that, despite substantial progress in this field, several important questions remain unresolved. To date, there is still no universally accepted recombinant antigen panel capable of simultaneously achieving high sensitivity, sufficient specificity, and reliable differentiation between post-vaccination and post-infection immune responses. In addition, serological outcomes are inevitably influenced by the stage of infection, prior vaccination history, and individual variability in humoral immune responses [70,73]. Collectively, these factors limit the direct translation of experimental findings into routine diagnostic practice.

Overall, the available evidence suggests that the most promising strategy is not the search for a single “optimal” antigen but rather the rational design of multicomponent antigen combinations. At present, this approach appears to be the most realistic and scientifically justified pathway for both the development of serological diagnostic systems and the design of subunit vaccines against orthopoxviruses. Future studies should therefore focus on the comparative evaluation of candidate antigens under standardized conditions, optimization of IMV and EV antigen combinations, and validation of their diagnostic performance using clinically characterized samples.

## 8. Conclusions

Current evidence indicates that the selection of recombinant antigens for orthopoxvirus serological diagnostics should take into account their structural organization, functional roles, and immunological characteristics. The high degree of antigenic conservation within the genus *Orthopoxvirus* underlies extensive cross-reactivity, which limits the utility of single-antigen approaches for differential diagnosis.

Analysis of the principal orthopoxvirus antigens demonstrated that M1, H3, and A29 play important roles in the induction of humoral immune responses and may serve as valuable targets for diagnostic applications, whereas A35 and B6 represent key antigens associated with the extracellular virion form.

Phylogenetic analyses further confirmed differences in the degree of protein conservation, which directly influences their diagnostic applicability. The most promising strategy appears to be the use of multicomponent antigen panels incorporating both IMV- and EV-associated proteins, thereby improving assay sensitivity while partially overcoming the limitations associated with serological cross-reactivity.

The findings summarized in this review provide a foundation for the development of next-generation serological diagnostic systems for orthopoxvirus infections. Future studies should focus on the experimental evaluation of the diagnostic potential of A29, M1, H3, A35, and B6 antigens under standardized conditions and using clinically characterized serum panels.

## Figures and Tables

**Table 1:** Natural reservoirs and zoonotic characteristics of orthopoxviruses.

Orthopoxvirus Species	Primary Reservoir	Incidental Hosts	Human Susceptibility	References
Macacapox virus (provisional designation)	Unknown; identified during an outbreak in macaques, while the natural reservoir remains undetermined	Non-human primates; domestic cats (sporadic cases)	Probable; isolated asymptomatic human infections have been reported	[2,13,14,15,16]
*O. akhmeta*	Unknown; likely small wild mammals, including rodents and shrews	Domestic livestock (cattle)	Yes (zoonotic cutaneous infections)	[17,18]
*O. camelpox*	Camels (*Camelus dromedarius*, *Camelus bactrianus*)	Not described	Yes (rare zoonotic infections, predominantly mild clinical forms)	[19,20]
*O. cowpox*	Small wild rodents (*Myodes glareolus*, *Microtus agrestis*, *Apodemus sylvaticus*)	Domestic cats, zoo animals, cattle	Yes (zoonotic)	[21,22,23]
*O. ectromelia*	House mouse (*Mus musculus*)	Not described	Human infection has not been documented	[24,25]
*O. monkeypox*	Not definitively established; African rodents, including rope squirrels, dormice, and pouched rats, are considered the most likely reservoirs	Non-human primates and multiple susceptible mammal species	Yes (major zoonosis) with documented human-to-human transmission	[26,27,28]
*O. raccoonpox*	Raccoons (*Procyon lotor*)	Cats	No confirmed natural human infections have been reported	[29,30,31]
*O. skunkpox*	Striped skunks (*Mephitis mephitis*)	Not described	Human infection has not been documented	[30]
*O. taterapox*	Presumed rodents (*Tatera kempi*); exact reservoir remains unknown	Not described	No human infections have been reported	[32,33]
*O. vaccinia*	Not definitively established	Evidence suggests circulation among multiple mammalian species in certain geographic regions	Yes (vaccination-associated, laboratory-acquired, and sporadic zoonotic infections)	[34]
*O. variola*	Humans are the only natural host and reservoir; no animal reservoir exists	None	Yes	[35,36]
*O. volepox*	Voles (*Microtus* spp., particularly *M. pennsylvanicus*)	*Peromyscus truei; Peromyscus californicus* shown to be experimentally susceptible	No confirmed human infections have been reported	[34,37]
*Borealpox virus*	Small mammals (primarily voles and shrews; exact reservoir remains under investigation)	Domestic cats, humans	Yes	[38,39]

**Table 2:** Orthopoxvirus sequences included in the phylogenetic analysis, with corresponding strains, accession numbers, and geographic origins.

Virus Species	Strain/Isolate	Accession Number	Geographic Origin
monkeypox virus	Congo_2003_358	DQ011154.1	Democratic Republic of the Congo
cowpox virus	Brighton Red	AF482758.2	United Kingdom
vaccinia virus	Copenhagen	M35027.1	Denmark
vaccinia virus	WR	AY243312.1	United States
horsepox virus	MNR-76	DQ792504.1	Mongolia
taterapox virus	Dahomey 1968	DQ437594.1	Benin
variola virus	India 1964 (Vellore)	DQ437586.1	India
camelpox virus	M-96	AF438165.1	Kazakhstan
camelpox virus	CMS	AY009089.1	Iran
rabbitpox virus	Utrecht	AY484669.1	Netherlands
ectromelia virus	Moscow	AF012825.2	Russia

**Table 3:** Structural, functional, and immunological characteristics of orthopoxvirus proteins (VACV and MPXV).

Gene (MPXV/VACV Ortholog)	Virion Form	Localization	Function	Predicted Molecular Weight (kDa)	Immunological Relevance	Role in Vaccine Design	References
M1R/L1R	MV/IMV	IMV membrane (entry-fusion complex-associated)	Mediates viral entry and membrane fusion; essential for IMV penetration into host cells	~25	Major target of IMV-neutralizing antibodies; recognized by monoclonal antibodies; exhibits broad cross-reactivity within the genus *Orthopoxvirus*	Widely incorporated into subunit and DNA vaccine platforms. Investigated as a target for therapeutic antibodies.	[48,95,96,97,98,99,100]
A35R/A33R	EV	Outer membrane of extracellular virion forms (IEV/CEV/EEV membranes)	Involved in morphogenesis and transport of extracellular virions; contributes to cell-to-cell viral spread and interacts with EV envelope proteins	23–28	Protective EV antigen: anti-A33 antibodies restrict viral dissemination and may exert complement-dependent neutralization	Frequently included in multivalent vaccine formulations combining MV and EV antigens.	[48,63,101,102]
B6R/B5R	EV	Surface membrane of EV particles	Critical for EV formation and dissemination; contains major neutralizing epitopes	~42	One of the principal EV-neutralizing antigens; antibody-mediated neutralization is often complement-dependent	Key EV component of vaccine formulations and antibody-based therapeutic cocktails; associated with broad protective activity.	[99,103,104,105,106]
H3L/H3L	MV/IMV	Surface membrane of IMV	Mediates attachment to host cells through heparan sulfate binding; important for infectivity	~35	Major target of neutralizing antibodies in humans; contributes substantially to protective immunity	MV antigen commonly included in multicomponent vaccines and serological assays.	[107,108,109,110,111]
E8L/D8L	MV/IMV	Surface membrane of IMV	Mediates attachment through chondroitin sulfate binding; participates in early stages of viral entry	~32	Highly immunogenic antigen: neutralizing monoclonal antibodies have been described; protective efficacy demonstrated for recombinant D8	Frequently combined with other MV antigens to broaden epitope coverage.	[105,112,113,114,115,116]
A29L/A27L	MV/IMV	Surface membrane of IMV	Mediates attachment to heparan sulfate; involved in virion assembly and egress; forms complexes with other structural proteins (including A26)	~14	Highly immunogenic; anti-A27 antibodies can neutralize viral infection, although protection may depend on the experimental model	Common MV component of multivalent vaccine platforms, including DNA vaccines.	[117,118,119,120]
A28L/A26L	MV/IMV (entry-fusion complex, EFC; also mediates entry of EV-derived IMV following outer membrane disruption)	IMV membrane. Component of the entry-fusion complex (EFC)	Entry-fusion complex subunit; essential for viral entry and membrane fusion	~16.3	Identified as a target of neutralizing antibodies; immunogenicity is enhanced in the context of the complex (e.g., with H2)	Potential MV antigen; also considered a functional target for studies of entry inhibitors.	[115,120,121,122,123]
F13L/F13L	EV-associated (IEV/CEV/EEV)	Cytoplasmic surface of wrapping membranes; associated with EV membranes (p37)	Required for IMV wrapping, IEV formation, and EV biogenesis; critical for viral egress; molecular target of tecovirimat	~37	Not considered a classical neutralizing antigen due to membrane topology, but essential for viral dissemination	Principal antiviral drug target (tecovirimat); also relevant for attenuation strategies and EV biogenesis studies.	[98,124,125,126,127]
K3L/(orthologs present in orthopoxviruses)	Non-structural protein.	Cytoplasm	PKR inhibitor acting as an eIF2α pseudosubstrate; contributes to host range determination	~10.5–11	Not typically associated with neutralizing antibody responses; major determinant of virulence and host adaptation	Classical target for viral attenuation and host adaptation studies; auxiliary target in vaccine vector engineering.	[128,129,130,131,132,133]
F3L/E3L	Non-structural protein	Predominantly cytoplasmic, with partial nuclear localization	Innate immune antagonist: binds dsRNA and inhibits IFN induction/ signaling pathways (including PKR, RNase L, and others)	~25 (p25)	Not considered a classical neutralizing antigen; central mediator of virulence and immune evasion	Common target for attenuation and safety optimization of vaccine strains; functional target for antiviral strategies.	[134,135,136]

## Data Availability

No new experimental data were generated in this study. All data analyzed during this work were obtained from publicly available databases and published literature, including GenBank accessioned sequences cited in the manuscript.

## Abbreviations

The following abbreviations are used in this manuscript:

A27	Orthopoxvirus intracellular mature virion surface protein A27
A29	Monkeypox virus intracellular mature virion surface protein A29
A33	Orthopoxvirus extracellular enveloped virion protein A33
A35	Monkeypox virus extracellular enveloped virion protein A35
B5	Orthopoxvirus extracellular enveloped virion glycoprotein B5
B6	Monkeypox virus extracellular enveloped virion glycoprotein B6
CPXV	Cowpox virus
EEV	Extracellular enveloped virion
ELISA	Enzyme-linked immunosorbent assay
EV	Extracellular virion
H3	Orthopoxvirus intracellular mature virion surface protein H3
IEV	Intracellular enveloped virion
IMV	Intracellular mature virion
L1	Orthopoxvirus intracellular mature virion membrane protein L1
MPXV	Monkeypox virus
mpox	Human monkeypox disease
mRNA	Messenger RNA
MV	Mature virion
NAAT	Nucleic acid amplification test
PCR	Polymerase chain reaction
VACV	Vaccinia virus
VARV	Variola virus
